# Repeat Intravitreal Dexamethasone Implant for Refractory Cystoid Macular Edema in Syphilitic Uveitis

**DOI:** 10.1155/2018/7419823

**Published:** 2018-02-19

**Authors:** Cassandra C. Lautredou, Joshua S. Hardin, John R. Chancellor, Sami H. Uwaydat, Abdallah A. Ellabban, Ahmed B. Sallam

**Affiliations:** ^1^College of Medicine, University of Arkansas for Medical Sciences, Little Rock, AR, USA; ^2^Jones Eye Institute, University of Arkansas for Medical Sciences, Little Rock, AR, USA; ^3^Department of Ophthalmology, Suez Canal University, Ismailia, Egypt

## Abstract

**Purpose:**

To report the successful utilization of adjunctive repeat intravitreal corticosteroid therapy for the treatment of cystoid macular edema in syphilis-related uveitis.

**Methods/Patients:**

An HIV-positive patient with treated ocular syphilis who developed refractory cystoid macular edema (CME) was treated with repeat intravitreal corticosteroid therapy including dexamethasone intravitreal implants.

**Results:**

Treatment led to the resolution of CME and improvement in visual acuity.

**Conclusions:**

Intravitreal corticosteroid therapy may be a viable adjunctive treatment for refractory CME in patients with treated syphilitic uveitis. Corticosteroid-induced exacerbation of infection is unlikely in patients with an adequate serologic treatment response.

## 1. Background

Ocular syphilis is characterized by inflammation of the eye due to* Treponema pallidum *infection. It may affect various ocular structures and manifest with anterior or posterior uveitis, vitritis, retinitis, Chorioretinitis, optic neuritis, and/or macular edema. Ocular syphilis presents most often in the secondary or tertiary stages of infection [1]. Its pathogenicity results from a combination of spirochete replication and immunologic response.

High dose intravenous penicillin remains the mainstay of treatment for ocular syphilis. Adjunctive corticosteroid therapy may decrease the severity of inflammation and prevent disease exacerbation after the initiation of antibiotic treatment, commonly termed the Jarisch-Herxheimer reaction. Topical corticosteroids have safely been used as an adjunctive treatment for syphilitic interstitial keratitis and anterior uveitis, while oral and intravenous corticosteroids are adjunctively utilized for treatment of syphilitic scleritis, posterior uveitis, and optic neuritis [2].

The use of intravitreal corticosteroids in the treatment of syphilis-related uveitis, however, is sparsely documented, due to clinical concern of syphilis reactivation after corticosteroid injection [3]. This concern mainly arises due to several reports that describe the unmasking or worsening of untreated ocular syphilis after intravitreal triamcinolone acetonide injection for what was presumed to be noninfectious, uveitis-related cystoid macular edema (CME) [4–6]. In this case report, we describe an HIV-positive patient with treated ocular syphilis who developed CME that was refractory to treatment but ultimately responded to repeat intravitreal corticosteroid therapy.

## 2. Case Report

A 42-year-old Caucasian male with a history of homosexual activity and intravenous drug use presented to our clinic with ocular pain and decreased vision in his right eye for several months. He had a new diagnosis of HIV with a CD4 count of 582 cells/mm^3^, 36% CD4+ T cells, and an HIV viral load of <20 copies/mL. At the time of diagnosis, syphilis IgG was also positive with a Rapid Plasma Reagin (RPR) titer of 1 : 128, indicating active infection. Cerebrospinal fluid studies demonstrated a nonreactive Venereal Disease Research Laboratory (VDRL) titer. On examination, his Snellen visual acuity (VA) was 20/100 in the right eye (OD) and 20/30 in the left eye (OS). Slit lamp examination revealed keratic precipitates, posterior synechiae of the iris, and 1+ cells and flare in the anterior chamber OD. Trace cells and aqueous flare were present in the anterior chamber OS. Funduscopic examination of the right eye revealed vitreous cells and 1+ vitreous haze, grade 2 disc edema, two patches of active retinitis in the inferior periphery, and pigmentary changes superiorly. Funduscopic examination of the left eye revealed trace vitreous cells and trace haze, with a normal optic disc, and two small foci of retinitis superonasally. There was no sign of CME clinically or on macular optical coherence tomography in either eye (OCT; Spectralis® device, Heidelberg Engineering GmbH, Germany).

**Figure 1:**
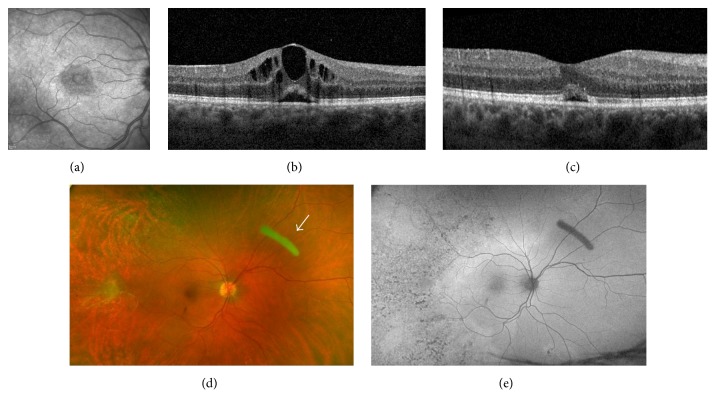
(a) Infrared fundus photo of the macula showing cystoid macular edema (CME); (b) optical coherence tomography (OCT) of the macula showing CME prior to treatment; (c) macular OCT showing resolution of CME with small amount of subretinal fluid; (d) color fundus photo after resolution of retinitis with white arrow pointing to the dexamethasone intravitreal implant; (e) autofluorescence fundus photo demonstrating temporal hypofluorescent changes that represent scarring following syphilitic retinitis.

The patient underwent treatment with intravenous penicillin G (24 million units/day for 14 days), along with topical prednisolone acetate 1% and oral prednisone (40 mg daily) as prophylaxis for a potential ocular Jarisch-Herxheimer reaction. On three-week follow-up, VA improved to 20/60 OD, with resolution of retinitis in both eyes and an improvement in disc edema OD. However, repeat OCT imaging revealed the presence of new CME in the right eye. Despite continued treatment, CME worsened over one month and vision decreased to 20/80 OD. Worsening of ocular symptoms led to concern for treatment failure, a known complication in HIV-positive patients. However, RPR titers were retested and had decreased to 1 : 16, consistent with a serologic treatment response. Macular edema persisted over the following 12 weeks despite treatment with three subsequent sub-Tenon's injections of triamcinolone acetonide (40 mg in 1 mL), a 4-week course of topical prednisolone acetate 1%, and nonsteroidal anti-inflammatory drops (Figures 1(a) and 1(b)). At this point, the decision was made to proceed with intravitreal corticosteroid therapy. We chose to start with intravitreal dexamethasone (400 *μ*g/0.1 mL) due to its short duration of action and utility as a therapeutic test. It resulted in mild improvement of CME over one week with no recurrence of retinitis. Subsequently, an intravitreal sustained-release dexamethasone implant (DEXA, 0.7 mg) was inserted. This treatment resulted in complete resolution of CME with improvement in VA to 20/30 OD (Figures 1(c)–1(e)). After three months, CME recurred with a reduction in vision necessitating a second dexamethasone implant. The patient responded once more to treatment. Throughout the follow-up period of 15 months, 4 intravitreal dexamethasone implants were inserted.

## 3. Discussion

We describe the successful utilization of repeat intravitreal corticosteroid therapy for refractory uveitic CME in the setting of treated ocular syphilis. Several reports have discussed the unmasking or worsening of untreated ocular syphilis after intravitreal triamcinolone acetonide injection for CME [4–6]. However, these reports describe patients who were neither diagnosed nor treated for syphilis prior to the intravitreal injection, indicating probable exacerbation of infection with local immunosuppression. The patient described in our report demonstrated a favorable treatment response to penicillin with an appropriate decrease in RPR titer prior to the use of intravitreal corticosteroids. However, CME occurred and was refractory to treatment with topical, periocular, and systemic corticosteroids.

Intravitreal corticosteroids may be the only effective treatment for refractory uveitic CME [6]. The caution exercised when using systemic corticosteroids in syphilis patients should also be applied when considering intravitreal corticosteroid treatment; such as was done in our patient, clinicians should continue to closely observe the eye for signs of retinitis reactivation and check RPR titers repeatedly to monitor for relapse or reinfection. Relapse is unlikely with successful treatment; however, reinfection is possible in patients engaging in high-risk sexual behavior [7].

To our knowledge there is only one case report in the literature where dexamethasone implant was used as adjuvant treatment for CME secondary to syphilitic uveitis [3]. However, only one implant was required in that case and there was no documentation of RPR titers showing that reinfection had been excluded prior to intravitreal steroid treatment [3].

In this report, we demonstrate that intravitreal dexamethasone implant may be a viable adjunctive treatment for refractory CME in patients with treated syphilitic uveitis. Corticosteroid-induced exacerbation of infection is unlikely in patients with an adequate serologic treatment response.
